# Early occlusion control of the intrapericardial inferior vena cava under femoral–femoral extracorporeal circulation using a technique to prevent pulmonary embolism during nephrectomy for renal cell carcinoma with tumor thrombus: two case reports

**DOI:** 10.1186/1756-0500-7-683

**Published:** 2014-10-01

**Authors:** Minoru Miyazato, Satoshi Yamashiro, Masato Goya, Hitoshi Inafuku, Akashi Ikehara, Yoshinori Oshiro, Seiichi Saito, Yukio Kuniyoshi

**Affiliations:** Department of Urology, Graduate School of Medicine, University of the Ryukyus, 207 Uehara, Nishihara, Okinawa 903-0215 Japan; Department of Thoracic and Cardiovascular Surgery, Graduate School of Medicine, University of the Ryukyus, Okinawa, 903-0215 Japan

**Keywords:** Intrapericardial inferior vena cava, Renal cell carcinoma, Tumor thrombus, Pulmonary embolism

## Abstract

**Background:**

Renal cell carcinoma with tumor thrombus extension into the inferior vena cava occurs in approximately 5% of cases. Despite such situations, an aggressive surgical approach is recommended. However, intraoperative prevention of pulmonary embolism by a fragmended tumor thrombus is necessary. To prevent pulmonary embolism, placement of a temporary suprarenal filter has been attempted, however, the precise placement of a temporary filter between the level of the hepatic vein and right atrium is not always easy because of its migration, tilting, and strut fracture. Here we report a method for early occlusion control of the intrapericardial inferior vena cava to prevent pulmonary embolism during nephrectomy in level II or III renal cell carcinoma tumor thrombus.

**Case presentation:**

Our first case was a 37-year-old Japanese man with left renal cell carcinoma extending into the inferior vena cava below the main hepatic vein (level II) and our second was a 75-year-old Japanese man with right renal cell carcinoma extending into the retrohepatic inferior vena cava at the main hepatic vein (level III). En block resection of the kidney and the tumor thrombus was performed with the aid of partial extracorporeal circulation; the postoperative course of both patients was uneventful.

**Conclusion:**

Control of intrapericardial inferior vena cava is a feasible method to prevent pulmonary embolism.

## Background

Renal cell carcinoma with tumor thrombus extension into the inferior vena cava (IVC) occurs in approximately 5% of cases [1]. Despite IVC invasion, an aggressive surgical approach for such neoplasms is recommended. However, intraoperative prevention of pulmonary embolism by a fragmended tumor thrombus is necessary. The frequency of a fatal pulmonary thrombus during such operations is 2–3.4% [2, 3]. To prevent perioperative fatal events, placement of a temporary suprarenal filter has been attempted with a favorable outcome [4]. However, complications of IVC filters include migration, tilting, and strut fracture [5]. It has been reported that intrapericardial control during IVC tumor thrombectomy is safe and feasible in such cases [6–8]. Here we present an approach regarding early occlusion of the suprahepatic IVC over the phrenic diagram under femoral–femoral extracorporeal circulation to prevent pulmonary embolism during nephrectomy for level II or III renal cell carcinoma tumor thrombus.

## Case presentation

### Case 1

**Figure 1:**
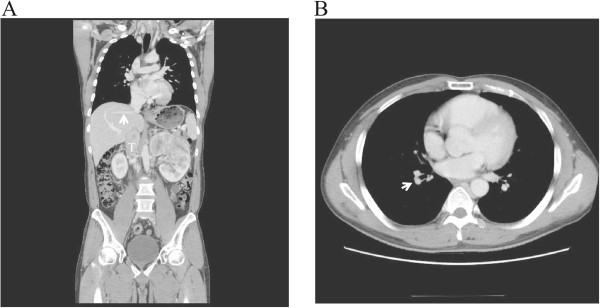
**Computed tomography scan of renal cell carcinoma with tumor thrombus (level II). (A)** Computed tomography scan of a 37-year-old man with left renal cell carcinoma showing a large tumor thrombus (T) extending into the retrohepatic inferior vena cava below the main hepatic vein (white arrow, level II). **(B)** A small embolism (T3bN0M1) of the right pulmonary artery (white arrow).

**Figure 2:**
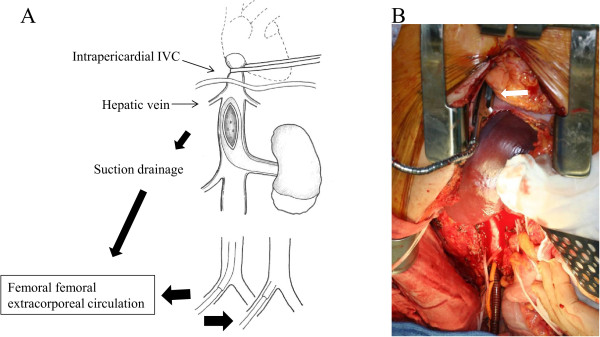
**Proposal technique. (A)** Early control of the intrapericardial inferior vena cava using vessel tape and removal of inferior vena cava tumor thrombus under femoral–femoral extracorporeal circulation. **(B)** The intrapericardial inferior vena cava was occluded with a clamp (white arrow) at the venacavotomy.

The patient was a 37-year-old Japanese man with a 10-cm left renal cell carcinoma extending into the retrohepatic IVC below the main hepatic vein (level II), as described by Naves and Zincke [9]. Since a small embolism of the right pulmonary artery was observed by computed tomography (T3bN0M1) (Figure 1), we were concerned about an increased risk of intraoperative mortality due to pulmonary embolism. Therefore, we first dissected and mobilized the kidney with the renal vein attached through a midline abdominal incision. Next, the midline incision was extended cranially into the sternum and the pericardium was incised. Then, the suprahepatic IVC was controlled intrapericardially using a vessel tape through a small window of the incised pericardium (Figure 2A). The intrapericardial IVC was occluded with a clamp just before partial extracorporeal circulation was initiated (Figure 2B). The femoral artery and vein were cannulated in the right groin. Under femoral–femoral extracorporeal circulation with an oxygenator (Figure 2A), a venacavotomy of approximately 10 cm at the level of the left renal vein enabled the removal of a 15-cm tumor thrombus. Finally, the venotomy was closed using a pericardium patch on the defective IVC wall that was resected due to tumor thrombus invasion. The left femoral arterial pressure was monitored during the cardiopulmonary bypass. Cardiopulmonary bypass flow was controlled by maintaining the left femoral arterial pressure to systemic pressure over 80 mmHg, consistent with our standard femoral–femoral bypass procedure. Radical nephrectomy was completed in 781 min with an estimated blood loss of 2067 ml, and the duration of partial extracorporeal circulation was 38 min. The patient’s early and late postoperative course was uneventful. The pathological diagnosis was papillary type II renal cell carcinoma (tumor size 10×9×7 cm, pT3bN2, Fuhrman grade 3).

### Case 2

**Figure 3:**
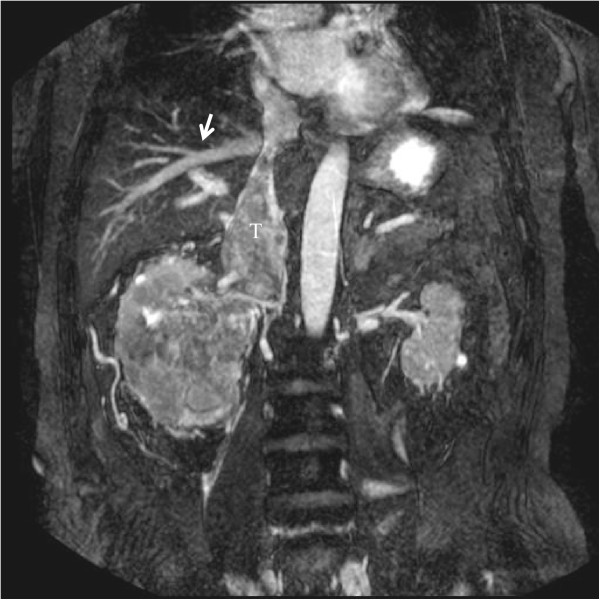
**Magnetic resonance images of renal cell carcinoma with tumor thrombus (level III).** Magnetic resonance images of a 75-year-old man with right renal cell carcinoma showing a large tumor thrombus (T) extending into the retrohepatic inferior vena cava at the main hepatic vein (white arrow, level III).

The patient was a 75-year-old Japanese man with a 9.5-cm right renal cell carcinoma extending into the retrohepatic IVC at the main hepatic vein (level III, T3bN0M0) (Figure 3). Through a thoracoabdominal incision over the 7th rib, the kidney was dissected and mobilized with the renal vein attached. The suprahepatic IVC was controlled using vessel tape and a clamp placed intrapericardially, as in Case 1. En block resection of the right kidney and tumor thrombus was performed during femoral–femoral extracorporeal circulation. The total surgical duration was 552 min with an estimated blood loss of 1840 ml, while the duration of partial extracorporeal circulation was 38 min. The patient was required open drainage for wound infection (Clavien grade 3a comorbidity) at the early postoperative course, but late postoperative course was uneventful. The pathological diagnosis was clear cell renal carcinoma (tumor size 9.6×8.3×4.5 cm, pT3bN0, Fuhrman grade 2).

## Discussion

Radical nephrectomy with thrombectomy remains the treatment of choice for renal cell carcinoma and an IVC tumor thrombus [2, 3]. The temporary placement of an IVC filter in the retrohepatic or suprahepatic segment to prevent pulmonary embolism is desirable [4]. However, the use of a temporary filter in a level II or III tumor thrombus is not always indicated because it is often difficult to precisely place a filter in the narrow space of IVC between the draining portion of the hepatic vein and right atrium. For such a case, occlusion control of the suprahepatic IVC via the intrapericardial approach is useful to prevent pulmonary embolism [6–8]. There are some important advantages to this method over the use of a temporary filter. First, occlusion of IVC can be performed regardless of the presence of an internal foreign body. Second, in terms of controlling the suprahepatic IVC, the intrapericardial IVC is easy to clamp because a posterior space can be straightforwardly created through a minimal incision to the pericardium compared with the precise placement of a temporary filter between the level of the hepatic vein and right atrium. Third, a pericardium patch can be applied to correct a defect of IVC wall resected due to tumor thrombus invasion, thus preventing IVC narrowing [10]. However, this approach may present some disadvantages, as it is more invasive because partial extracorporeal circulation is necessary, although the duration of partial extracorporeal circulation was very short. Hence, this technique seems very safe.

Various surgical maneuvers have been suggested based on thrombus extent or personal preference [11]. In our patient, a midline left-side incision including sternotomy was chosen with a thoracoabdominal approach to the right side. The use of both approaches achieved better exposure and early control of the intrapericardial IVC. Although the usefulness of extracorporeal circulation in patients with a level IV thrombus is obvious, its routine use in those with a level II or III thrombus is not compulsory [8]. However, in our patient, partial extracorporeal circulation via the femoral artery to the femoral vein was helpful to minimize blood loss [10].

## Conclusion

Our proposed technique is a feasible method to control the inferior vena cava for renal cell carcinoma extending into IVC below or at the level of the hepatic vein (level II or III).

## Consent

Written informed consent was obtained from both patients for publication of this Case Report and any accompanying images. A copy of the written consent is available for review by the Editor-in-Chief of this journal.

## Abbreviations

IVC: Inferior vena cava.
